# Pharmacokinetics and safety profiles of beinaglutide injection, a recombinant human GLP-1, in adults with overweight/obesity: results from a phase I clinical trial

**DOI:** 10.3389/fphar.2024.1433587

**Published:** 2024-08-22

**Authors:** Pingping Lin, Chengqian Li, Yanping Liu, Feifei Sun, Tsung-Han Hsieh, Lin Zhang, Yaping Ma, Xiaomeng Gao, Qing Yu, Yu Cao

**Affiliations:** ^1^ Clinical Trials Center, The Affiliated Hospital of Qingdao University, Qingdao, Shandong, China; ^2^ Department of Endocrinology and Metabolism, The Affiliated Hospital of Qingdao University, Qingdao, Shandong, China; ^3^ Shanghai Benemae Pharmaceutical Corporation, Shanghai, China

**Keywords:** beinaglutide, glucagon-like peptide 1, overweight, obesity, pharmacokinetics

## Abstract

**Background:**

Beinaglutide, whose active ingredient is rhGLP-1, has been widely used as a pharmacological therapy for T2DM. We explored the safety and pharmacokinetics of beinaglutide in Chinese overweight/obese volunteers to lay a foundation for clinical applications of beinaglutide as an anti-obesity drug.

**Methods:**

An open-label, single center, multiple ascending dose phase I clinical trial was conducted in 16 overweight/obese Chinese volunteers. The plasma concentrations of beinaglutide were determined by a validated ELISA method and the pharmacokinetic parameters were estimated via non-compartmental analysis methods. Adverse events were also recorded.

**Results:**

Beinaglutide sequentially multiple dosing (three times daily) at different doses were generally well tolerated, without serious AEs leading to discontinuation of the trial. After multiple subcutaneous injections of different doses (0.1, 0.14 and 0.2 mg), the average blood concentration of beinaglutide with or without baseline correction showed a similar trend among different dose groups on different study days. After reaching the peak concentration around 15 min, it began to decrease, and the median of T_max_ and T_max,adj_ was 10–15 min. The exposure *in vivo* increased in proportion to the dosage increment, demonstrating linear pharmacokinetic characteristics. There were no statistically significant differences in the main PK parameters and no accumulation of beinaglutide after multiple dosing. After multiple subcutaneous injections, a gender difference was observed, while no differences in BMI were found under the grouping conditions.

**Conclusion:**

The safety profile and pharmacokinetic properties support further development and clinical applications of beinaglutide as an anti-obesity drug.

**Systematic Review Registration:**

[https://register.clinicaltrials.gov/prs/app/action/SelectProtocol?sid=S000BPEI&selectaction=Edit&uid=U00050YQ&ts=2&cx=wy0ioj].

## 1 Introduction

Overweight/obesity is increasing worldwide and significantly increases the risk of heart disease, stroke, type 2 diabetes (T2DM), cancer, osteoarthritis and many other diseases greatly, leading to a substantial financial burden on our healthcare system (Danaei et al., 2011; Blüher, 2019). Therefore, overweight/obesity is a vast public health problem, and the prevention and control of which has become an urgent task. Despite the existence of several anti-obesity drug candidates, the target populations are deterred by their limited efficacy or unacceptable adverse effects (Adan, 2013; Scheen and Van Gaal, 2014).

Glucagon-like peptide 1 (GLP-1), mainly secreted from gut enteroendocrine cells, controls meal-related glycemic excursions through stimulation of insulin secretion and inhibition of glucagon secretion. As an incretin hormone, GLP-1 are widely used as pharmacological therapies for type-2 diabetes mellitus. In addition to regulating bloodglucose, GLP-1 reduces inflammation and apoptosis, improving cardiovascular function and neuroprotection (Müller et al., 2019). Recent studies have revealed that GLP-1 and its analogs can also reduce appetite and lose weight by inhibiting gastric emptying and feeding centers to feel satiety (Nadkarni et al., 2014; Zhang et al., 2019). Beinaglutide injection, also known as recombinant human GLP-1 (7-36) [rhGLP-1 (7-36)], was obtained through genetic engineering technology, whose active ingredient has the same amino acid sequence as GLP-1 in the human body (Wang et al., 2021). As a humanized GLP-1, beinaglutide has the same mechanism of action as the active ingredient of human GLP-1. The drug was approved by the China National Medical Products Administration (NMPA) for the treatment of T2DM in December 2016.

In previous phase 2 and 3 clinical trials of beinaglutide for the treatment of T2DM, the most common adverse reactions in subjects treated with 0.1 mg and 0.2 mg were mild to moderate nausea (Center for drug evaluation and NMPA). The aforementioned adverse reactions mostly occurred within 30 min after administration and resolved in approximately 1 to 2 h (Center for drug evaluation and NMPA). With the frequency and severity of symptoms decreasing as the treatment duration extended. Other adverse reactions included dizziness, fatigue, and vomiting. There have been no reports of acute pancreatitis or thyroid-related adverse events associated with beinaglutide, as seen with other GLP-1 receptor agonists (GLP-1RAs) (Center for drug evaluation and NMPA). A real-world safety surveillance also showed that nausea, dizziness and appetite loss were the most frequently occurred adverse events (AEs) in T2DM patients treated with beinaglutide (Shanghai benemae pharmaceutical corporation, 2023), which is consistent with previous clinical studies.

The pharmacokinetic (PK) study of benaglutide in healthy subjects exhibited linear PK characteristics in the dose range of 0.1 to 0.2 mg. There is no accumulation of beinaglutide after multiple injection when subjects were subcutaneously injected with beinaglutide three times a day for five consecutive days (Shanghai benemae pharmaceutical corporation, 2023). Upon subcutaneous injection of 0.2 mg of beinaglutide in healthy subjects, absorption is rapid, with plasma drug concentrations peaking at 19 min, reaching a peak concentration of 642 ng/L. The area under the blood concentration-time curve from time zero to infinity (AUC_0-inf_) is calculated to be 19,687 ng·L^-1^·min. Distribution of the drug is extensive, with an apparent volume of distribution (V_z_/F) observed to be 379 L in healthy subjects (Center for drug evaluation and NMPA). In terms of metabolism and elimination, beinaglutide has a half-life of approximately 11 min, indicating its rapid and complete metabolism within the body without any accumulation. Additionally, beinaglutide is primarily excreted through the urine (Center for drug evaluation and NMPA).

As there are currently limited studies evaluating beinaglutide in subjects other than T2DM patients, we herein conducted this study to explore the safety and PK characteristics of beinaglutide in Chinese volunteers with overweight/obesity, and lay a foundation for clinical applications of beinaglutide as an anti-obesity drug.

## 2 Materials and methods

### 2.1 Investigational drug

Beinaglutide injection [rhGLP-1 (7-36), 2.1 mL: 4.2 mg, manufactured by Shanghai Benemae Pharmaceutical Corporation] was used in this study. The study drugs were pen-type syringe, packaged with neutral borosilicate glass sleeve before used, sealed at 2°C–8°C and stored away from light. After the first use, it can be stored at the temperature not higher than 25°C for 2 weeks, and stored at 2°C–8°C for 6 weeks.

### 2.2 Compliance with ethics guidelines

The study protocol was approved by the Medical Ethics Committee at the Affiliated Hospital of Qingdao University. All procedures were performed in compliance with the Declaration of Helsinki (World Medical Association, 2013) as well as the International Conference on Harmonization Guideline for Good Clinical Practice (Switula, 2000). All volunteers provided written informed consent before being screened for eligibility. This study was registered with the Clinical Trial Registry (trial ID: NCT05226000).

### 2.3 Study participants

Adult male or female subjects were eligible for participation if they were aged 18–70 years with a body mass index (BMI) meeting one of the following requirements (a or b): a. 28 kg/m^2^ or greater, b. 24 kg/m^2^ or greater, and accompanied by at least one of the following manifestations: vigorous appetite, unbearable hunger before meals, and large intake each meal; combined with one or several of the following: hyperglycemia, hypertension, dyslipidemia, fatty liver; combined with weight-bearing joint pain; combined with obesity-related dyspnea or obstructive sleep apnea syndrome. Additionally, subjects were required to be deemed in good health by the investigator based on medical history, vital signs, physical examination, 12-lead electrocardiogram (ECG), chest X-ray, abdominal color Doppler ultrasonography and clinical laboratory tests (blood routine, urine routine, blood biochemistry, virus test, pregnancy test for female, etc.). Subjects were excluded from the study if they met any of the following criteria: subjects explicitly diagnosed with type 1 or type 2 diabetes. Additionally, those who had weight change exceeding 5% in the 3 months prior to screening, used weight gain drugs, weight loss drugs or hypoglycemic drugs that may affect body weight within 4 weeks prior to screening were excluded. Subjects who had used any prescription, over-the-counter, vaccine products, or herbal medicine during the 14 days prior to the start of the trial, or had heart disease, active liver disease, kidney damage, moderate or severe gastrointestinal diseases combined with gastrointestinal motility disorders or obstructive intestinal diseases were excluded from the study. Female subjects who were breastfeeding or pregnant were ineligible for participation.

### 2.4 Study design

**FIGURE 1:**
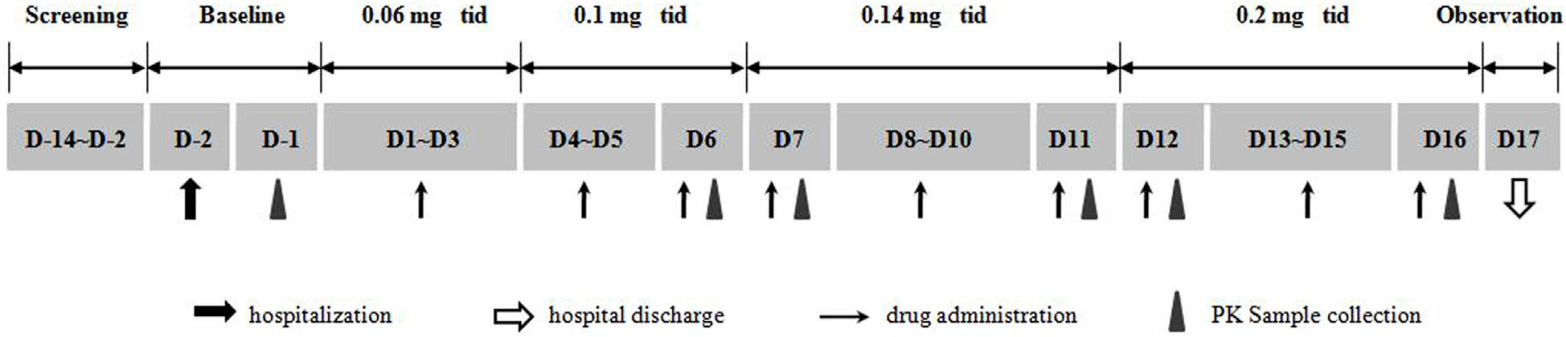
Study design.

This single center, open, multiple ascending dose study was conducted at the Phase I Clinical Research Center of the Affiliated Hospital of Qingdao University, Qingdao, China. Adult subjects with overweight or obesity were enrolled, and each of them sequentially received multiple dosing (three times daily) of beinaglutide at different doses of 0.06, 0.1, 0.14, and 0.2 mg, respectively. The duration of each dose was 0.06 mg for three consecutive days, 0.1 mg for three consecutive days, 0.14 mg for five consecutive days, and 0.2 mg for five consecutive days (Figure 1). All volunteers were hospitalized 2 days before the first dosing and placed on a standardized diet during hospitalization. All participants were required to fast overnight before the first dosing. Different doses of beinaglutide were subcutaneously injected 5 min before each meals every day for 16 days.

### 2.5 Blood sampling and assay methods

Blood samples (3 mL) were collected in vacuum tubes containing EDTA-K_2_ at D-1 and at pre-dosing (0 h) and 5, 10, 15, 20, 25, 30, 40, 50, 60, 90, 120, 150 and 180 min after first administration on D6, D7, D11, D12, and D16. PK blood samples were collected at the time point corresponding to the administration date for blank baseline correction on D-1. The vacuum tubes were pre-cooled in the refrigerator (2°C–8°C) for at least 0.5–1 h before use. As the stabilizer, 30 μL DPP IV inhibitor (10 μL/mL) was immediately added into the blood. The stabilizer-spiked blood samples were kept with wet ice during transportation and were centrifuged at 3,000 rpm for 10 min at 4°C to separate the plasma. Samples were stored at −80°C until analysis.

The concentrations of beinaglutide in plasma samples were determined at Teddy Clinical Research Laboratory Limited (Shanghai, China), and analyzed by the ELISA method, which was validated in terms of accuracy, precision, durability, the matrix effect, dilution linearity and HOOK effect, specificity, and stability. The standard curve ranged from 2.0 to 100.0 pM. The accuracy of intra-batch and inter-batch assays values were −16.7%–20.0% and −6.7%–3.2%, respectively. The precision of intra-batch and inter-batch assays values were 0.5%–16.7% and 6.9%–15.0%, respectively. The anti-matrix interference ability, dilution linearity, specificity and stability of this method all met the detection requirements of beinaglutide.

### 2.6 Safety assessment

Safety assessments were conducted by monitoring physical examinations, vital signs (blood pressure, pulse rate, body temperature and respiratory rate), 12-lead ECG, clinical laboratory assays (blood routine, blood biochemistry and urine routine) and AEs. AEs were evaluated by the investigator throughout the study and the severity of AEs was defined in accordance with the Common Terminology Criteria for the Classification of Adverse Events of the National Cancer Institute (NCI CTCAE) version 5.0.

### 2.7 Pharmacokinetic analysis

The primary PK parameters were maximum observed concentration (C_max_), the area under concentration-time curve from 0 to the last measurable concentration (AUC_0-t_) and AUC_0-inf_, whereas the secondary PK parameters were median time to reach maximum concentration (T_max_), terminal half-life time (t_1/2_), V_z_/F, apparent clearance rate (CL/F), terminal elimination rate (λ_z_) and accumulation index (R_ac_).

The baseline-adjusted concentration was calculated as the difference between beinaglutide concentration at the selected time point on D1 and the concentration at the corresponding time point on D-1. Correspondingly, the baseline-adjusted PK parameters were calculated based on the baseline-adjusted concentration of beinaglutide.

### 2.8 Statistical analysis

All enrolled subjects were analyzed for demographic data and baseline characteristics, etc. All enrolled subjects who received at least one injection of the study drug were evaluated for tolerability. All volunteers received beinaglutide who had at least one measurable PK concentration and no scheme deviation that had a significant impact on PK indexes (C_max_, AUC, etc.) were included in PK analysis. PK parameters were calculated from the plasma concentration data of beinaglutide by non-compartmental analysis (NCA) using Phoenix WinNonlin (version 8.3.1). Paired t-tests were used to compare the primary PK parameters between the 0.14 mg and 0.2 mg dose groups. Three doses (0.1, 0.14 and 0.2 mg) were evaluated for proportional dose-response relationships using power model. To evaluate potential gender or BMI-related differences in the PK profile of beinaglutide, analysis of variance (ANOVA) or independent sample T tests were performed on the main PK parameters (C_max_, AUC_0-t_, AUC_0-inf_ without baseline correction, and C_max, adj_, AUC_0-t, adj_, AUC_0-inf, adj_) of beinaglutide following multiple doses (0.1, 0.14, and 0.2 mg) administered on D6, D11 and D16, respectively, across different gender or BMI groups. Statistical analysis was performed using SAS 9.4 (SAS Institute, Cary, NC, United States).

## 3 Results

### 3.1 Subject disposition and characteristics

Eighty subjects were screened, 16 were randomized, and 14 completed the trial (Table 1). 68.8% of the enrolled subjects were male while 31.3% were female, and their ages ranged from 19 to 42 years old. All 16 exposed subjects were included in the safety analysis set, the full analysis set and the PK analysis set. Subject characteristics overall are shown in Table 1.

**TABLE 1:** Demographics and baseline characteristics.

Characteristics	Beinaglutide injection (n = 16)	
Age (y)		
	Mean ± SD	30.0 ± 6.4
	Range	19.0–42.0
Gender (n)		
	Male	11 (68.8%)
	Female	5 (31.3%)
Nationality (n)		
	Han	15 (93.8%)
	Other	1 (6.3%)
Height (cm)		
	Mean ± SD	169.9 ± 6.2
	Range	159.0–186.0
Empty body weight (kg)		
	Mean ± SD	92.7 ± 13.4
	Range	70.6–119.3
BMI (kg/m^2^)		
	Mean ± SD	32.1 ± 4.8
	Range	25.6–41.6

### 3.2 Safety

All enrolled subjects completed the medication plan of 0.06 mg and 0.1 mg for three consecutive days, and 0.14 mg for five consecutive days. Fourteen subjects completed the regimen of 0.2 mg for five consecutive days, while two subjects withdrew prematurely due to self-reported intolerance, specifically nausea and vomiting, after dosage adjustment to 0.2 mg. One subject completed 7 doses of 0.2 mg medication, while the other subject completed 3 doses of 0.2 mg before withdrawal. Blood samples after D12 were not collected from the two subjects who withdrew early.

A total of 15 subjects (93.8%, 15/16) experienced at least one AE, with a total of 243 reports. Among these reports, 209 reports were determined to be drug-related (Table 2). The most common reported drug-related treatment emergent adverse events (TEAEs) included nausea (97 reports, 43.8%, 7/16), dizziness (67 reports, 31.3%, 5/16), and urine ketone body present (5 reports, 31.3%, 5/16) and there was no significant difference between dose groups. Nausea had a higher incidence during the high-dose injection phase but could be relieved within about 1 h. All AEs were mild in severity. There were no AEs associated with the study drug that resulted in discontinuation of the trial and no serious AEs or serious adverse reactions observed.

**TABLE 2:** Summary of AEs.

	Beinaglutide														
	0.06 mg			0.1 mg			0.14 mg			0.2 mg			Total		
	N = 16			N = 16			N = 16			N = 16			N = 16		
	E	n	%	E	n	%	E	n	%	E	n	%	E	n	%
AEs	7	5	31.3%	12	7	43.8%	42	9	56.3%	182	11	68.8%	243	15	93.8%
Drug-related AEs	1	1	6.3%	2	2	12.5%	34	5	31.3%	172	8	50.0%	209	11	68.8%
Serious AEs	0	0	0.0%	0	0	0.0%	0	0	0.0%	0	0	0.0%	0	0	0.0%
Drug-relatedseriousAEs	0	0	0.0%	0	0	0.0%	0	0	0.0%	0	0	0.0%	0	0	0.0%
AEs leading to discontinuation of drug	0	0	0.0%	0	0	0.0%	0	0	0.0%	0	0	0.0%	0	0	0.0%
Drug-related AEs leading to discontinuation of drug	0	0	0.0%	0	0	0.0%	0	0	0.0%	0	0	0.0%	0	0	0.0%
AEs leading to death	0	0	0.0%	0	0	0.0%	0	0	0.0%	0	0	0.0%	0	0	0.0%
Drug-related AEs leading to death	0	0	0.0%	0	0	0.0%	0	0	0.0%	0	0	0.0%	0	0	0.0%
Severity															
Severe	0	0	0.0%	0	0	0.0%	0	0	0.0%	0	0	0.0%	0	0	0.0%
Moderate	0	0	0.0%	0	0	0.0%	0	0	0.0%	0	0	0.0%	0	0	0.0%
Mild	7	5	31.3%	12	7	43.8%	42	9	56.3%	182	11	68.8%	243	15	93.8%
Outcome															
Recovered/Recovering	6	5	31.3%	9	5	31.3%	40	8	50.0%	172	9	56.3%	227	13	81.3%
Unknown	1	1	6.3%	3	2	12.5%	2	1	6.3%	10	6	37.5%	16	7	43.8%
Drug-related TEAEs with higher occurrence															
Nausea	0	0	0.0%	0	0	0.0%	15	1	6.3%	82	7	43.8%	97	7	43.8%
Dizziness	0	0	0.0%	0	0	0.0%	15	1	6.3%	52	5	31.3%	67	5	31.3%
Urine ketone body present	0	0	0.0%	2	2	12.5	3	3	18.8%	0	0	0.0%	5	5	31.3%

N: total number of subjects.

E: number of adverse events.

n: number of subjects with at least one adverse event.

%: percentage of subjects with at least one adverse event.

### 3.3 Pharmacokinetics

**FIGURE 2:**
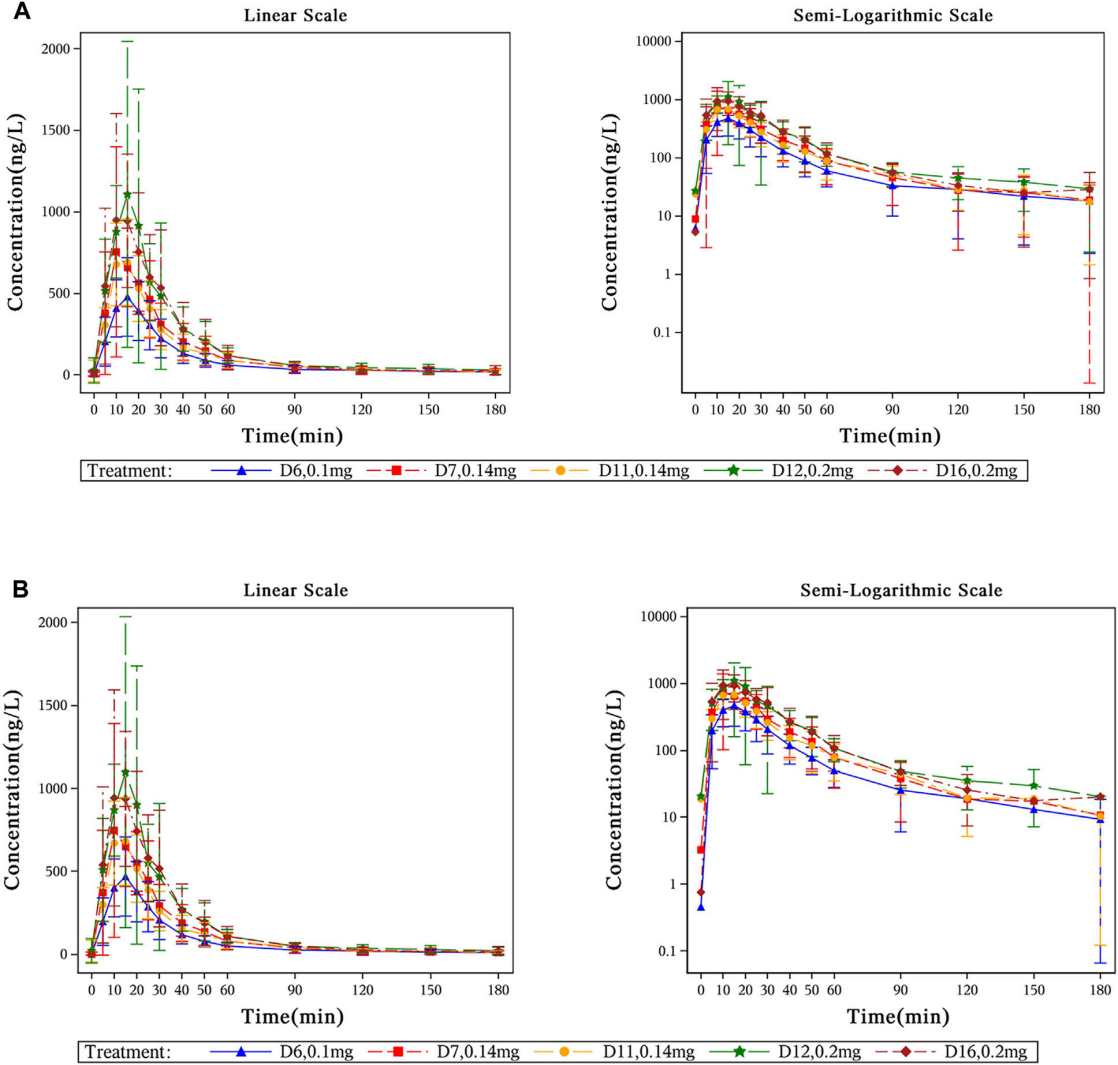
Mean plasma concentration-time profiles of beinaglutide in adult subjects with overweight/obesity after multiple subcutaneous injections. **(A)** Mean plasma concentration-time curves of beinaglutide without baseline correction in adult subjects with overweight/obesity after multiple subcutaneous injections (in linear scale and in semi-logarithmic scale). **(B)** Mean plasma concentration-time curves of beinaglutide with baseline correction in adult subjects with overweight/obesity after multiple subcutaneous injections (in linear scale and in semi-logarithmic scale).

The mean plasma concentration-time profiles of beinaglutide in adult subjects with overweight/obesity after multiple subcutaneous injections of different doses were presented in Figure 2 (baseline unadjusted or adjusted), and its derived PK characteristics (baseline adjusted) were summarized in Table 3. After multiple subcutaneous injections of different doses (0.1, 0.14 and 0.2 mg) of beinaglutide in adult subjects with overweight/obesity, the median of T_max,adj_ was 10 to 15 min, while the arithmetic mean of C_max,adj_, AUC_0-t,adj_ and AUC_0-inf,adj_ were 485.55–1,183.55 ng/L, 14646.13–32992.45min*ng/L and 15437.81–34586.63 min*ng/L, respectively. It can be recognized that the exposure of the drug increased with the increased dosage. Over the range of 0.1 mg to 0.2 mg, the arithmetic mean values of t_1/2, adj_, CL/F_adj_, and V_z_/F_adj_ were 29.64–42.35 min, 6.81–7.66 L/min and 292.55–401.87 L, respectively. The arithmetic mean values of R_ac_Cmax, adj_ under the dosage of 0.14 mg and 0.2 mg were 1.05 and 1.04, respectively, while that of R_ac_AUC, adj_ under the same dosage were 1.06 and 0.99, respectively, revealing that there is no accumulation of beinaglutide after multiple injection *in vivo*.

**TABLE 3:** Summary of pharmacokinetic parameters for beinaglutide after multiple administration (baseline adjusted).

PK parameters	Dose group				
	0.1 mg	0.14 mg		0.2 mg	
	D6 (n = 16)	D7 (n = 16)	D11 (n = 16)	D12 (n = 16)	D16 (n = 14)
C_max, adj_ (ng/L)	485.55 ± 233.02	813.22 ± 624.74	728.85 ± 257.41	1,183.55 ± 918.45	1,099.40 ± 638.29
T_max, adj_ (min)	15.00 (10.00–20.00)	15.00 (5.00–20.00)	15.00 (10.00–15.00)	10.00 (5.00–20.00)	15.00 (5.00–30.00)
AUC_0-t, adj_ (min^a^ng/L)	14646.13 ± 6612.60	22741.77 ± 11403.58	21245.12 ± 7596.82	32992.45 ± 17873.34	31771.40 ± 14702.51
AUC_0-inf, adj_ (min^a^ng/L)	15437.81± 6609.42	23657.68± 11729.14	21935.02± 7832.23	34586.63± 18930.02	33392.48± 14345.88
CL/F_adj_ (L/min)	7.66 ± 3.09	7.39 ± 3.45	7.08 ± 2.20	6.81 ± 2.40	7.25 ± 3.38
V_z_/F_adj_ (L)	356.63 ± 235.35	297.46 ± 120.01	292.55 ± 105.75	401.87 ± 225.34	352.11 ± 199.97
t_1/2, adj_ (min)	32.65 ± 16.83	30.63 ± 10.75	29.64 ± 9.26	42.35 ± 22.02	34.44 ± 16.11
λ_z, adj_ (1/min)	0.03 ± 0.01	0.03 ± 0.01	0.03 ± 0.01	0.02 ± 0.01	0.02 ± 0.01
R_ac_Cmax, adj_ ^a^	—	—	1.05 ± 0.33	—	1.04 ± 0.50
R_ac_AUC, adj_ ^a^	—	—	1.06 ± 0.40	—	0.99 ± 0.21

Data are shown as arithmetic mean ± SD, except T_max_, which is median (range).

^a^ only the result of the last dose of multiple dosing was calculated.

There was no significant difference in primary PK parameters between multiple injections of 0.14 mg and 0.2 mg of beinaglutide in overweight/obesity subjects. Table 4 showed the results of dose proportional response relationship analysis. In the range of 0.1 mg–0.2 mg, the primary PK parameters, both baseline unadjusted (C_max_, AUC_0-t_ and AUC_0-inf_) and adjusted (C_max,adj_, AUC_0-t,adj_ and AUC_0-inf,adj_), were positively proportional to dose increase, showing linear PK characteristics of beinaglutide.

**TABLE 4:** Proportional dose-response relationship of primary PK parameters after multiple administration of beinaglutide

	PK parameters	n	Estimate of slope β1	90% CI
Baseline unadjusted				
	Ln (C_max_)	78	1.13	(0.79, 1.47)
	Ln (AUC_0-t_)	78	1.04	(0.72, 1.36)
	Ln (AUC_0-inf_)	78	1.01	(0.68, 1.34)
Baseline adjusted				
	Ln (C_max,adj_)	78	1.16	(0.81, 1.50)
	Ln (AUC_0-t,adj_)	78	1.13	(0.82, 1.44)
	Ln (AUC_0-inf,adj_)	78	1.12	(0.82, 1.43)

### 3.4 Gender differences analysis

Independent sample t-test results for gender grouping using dose normalized logarithmic conversion of the main PK parameters (C_max_, AUC_0-t_, AUC_0-inf_ without baseline correction, and C_max, adj_, AUC_0-t, adj_, AUC_0-inf, adj_) of beinaglutide after multiple doses (0.1, 0.1 and 0.2 mg) administered (D6, D11 and D16) were shown in Table 5. The results showed there is a gender difference in the exposure of beinaglutide in adults with overweight/obesity, and the exposure level of female subjects is higher than that of male subjects.

**TABLE 5:** Analysis of PK parameters of beinaglutide in plasma in subjects of different gender grouping.

	Gender	Statistics	PK parameters		
			Ln (C_max_/Dose) (ng/L/mg)	Ln (AUC_0-t_/Dose) (min*ng/L/mg)	Ln (AUC_0-inf_/Dose) (min*ng/L/mg)
Baseline unadjusted					
		n	32	32	32
		Mean	8.30	11.78	11.87
	male	SD	0.42	0.40	0.41
		GeoMean	8.29	11.77	11.86
		%CV_b_	5.04	3.38	3.43
		n	14	14	14
		Mean	8.84	12.26	12.34
	female	SD	0.24	0.31	0.30
		GeoMean	8.84	12.26	12.34
		%CV_b_	2.66	2.53	2.49
		*p* value	<0.001	<0.001	<0.001
Baseline adjusted					
		n	32	32	32
		Mean	8.29	11.70	11.76
	male	SD	0.43	0.36	0.35
		GeoMean	8.28	11.70	11.76
		%CV_b_	5.21	3.10	2.94
		n	14	14	14
		Mean	8.82	12.18	12.21
	female	SD	0.24	0.34	0.34
		GeoMean	8.82	12.18	12.20
		%CV_b_	2.72	2.86	2.86
		*p* value	<0.001	<0.001	<0.001

### 3.5 BMI differences analysis

Table 6 showed variance analysis results for BMI differences (two classifications, BMI> 28 and BMI≤ 28, respectively) using the main PK parameters (C_max_, AUC_0-t_, AUC_0-inf_ without baseline correction, and C_max, adj_, AUC_0-t, adj_, AUC_0-inf, adj_) after logarithmic conversion of beinaglutide after multiple doses (0.1, 0.14, and 0.2 mg) administered (D6, D11 and D16). The *p* values compared between the BMI >28 and BMI ≤28 groups were greater than 0.05, indicating no statistically significant difference between the two groups based on BMI classification.

**TABLE 6:** Analysis of main PK parameters of beinaglutide in plasma of different BMI subjects (two classifications).

	Parameters	BMI (kg/m^2^)	n	GeoLSM	Classifications	Ratio (%)	90% CI of ratio (%)	*p* value
Baseline unadjusted								
	C_max_/Dose (ng/L/mg)	>28	34	4309.62	BMI> 28 vs. BMI≤ 28	66.54	(46.22, 95.78)	0.069
		≤28	12	6476.99				
	AUC_0-t_/Dose (min*ng/L/mg)	>28	34	139565.11	BMI> 28 vs. BMI≤ 28	69.68	(46.80, 103.76)	0.132
		≤28	12	200284.77				
	AUC_0-inf_/Dose (min*ng/L/mg)	>28	34	153601.21	BMI> 28 vs. BMI≤ 28	71.64	(47.58, 107.87)	0.173
		≤28	12	214415.96				
Baseline adjusted								
	C_max_/Dose (ng/L/mg)	>28	34	4235.84	BMI> 28 vs. BMI≤ 28	66.48	(45.89, 96.33)	0.073
		≤28	12	6371.27				
	AUC_0-t_/Dose (min*ng/L/mg)	>28	34	129638.21	BMI> 28 vs. BMI≤ 28	72.54	(49.40, 106.51)	0.163
		≤28	12	178719.58				
	AUC_0-inf_/Dose (min*ng/L/mg)	>28	34	137654.63	BMI> 28 vs. BMI≤ 28	74.84	(51.77, 108.19)	0.188
		≤28	12	183927.36				

## 4 Discussion

Based on evidence of randomized controlled trial, a previous study (Shi et al., 2022) conducted systematic evaluation and network meta-analysis on eight types of weight control drugs, which showed that in adults with overweight or obesity, phentermine-topiramate and GLP-1RAs proved the best drugs in reducing weight. In addition, multiple studies (Nadkarni et al., 2014; Zhang et al., 2019; Wang et al., 2021) have shown that beinaglutide may significantly reduce the weight of T2DM patients by delaying gastric emptying and reducing food intake. As the only GLP-1RAs identical to human GLP-1 (7–36), beinaglutide is expected to provide a new treatment option for the prevention and management of overweight/obesity and become the first novel GLP-1RA approved for weight management indications in China. Therefore, we conducted this study to explore the safety and pharmacokinetics of beinaglutide in Chinese volunteers with overweight/obesity.

It is worth noting that the properties and incidence of gastrointestinal AEs (such as nausea and vomiting) after treatment with beinaglutide are similar to those in previous studies such as phase 2, phase 3 studies of beinaglutide in the treatment of T2DM and the phase 3 study of beinaglutide for weight management in Chinese individuals with overweight/obesity (Center for drug evaluation and NMPA; Torekov et al., 2011; Ambery et al., 2018; Elbrønd et al., 2002; Chen et al., 2024). The most frequently occurred gastrointestinal AE was nausea that primarily occurred during the dose-escalation period, and was mild in severity and transient. This may be associated with the pharmacological effects of GLP-1RAs. Moreover, the treatment discontinuation rate (12.5%, 2/16) due to self-reported intolerance of nausea and vomiting was acceptable. The results of safety evaluations, including AEs, clinical laboratory examinations, vital signs, electrocardiogram, and physical examinations, indicated that multiple subcutaneous injections of beinaglutide within the dose range of 0.06 mg to 0.2 mg were safe and tolerable.

After multiple subcutaneous injections of different doses of beinaglutide in adult subjects with overweight/obesity, T_max_, t_1/2_ and CL/F remain basically unchanged over the dosage range of 0.1 mg to 0.2 mg. The exposure levels of beinaglutide (AUC_0-t_ and C_max_) was positively proportional to the increase of dosage, exhibiting linear PK characteristics. After multiple subcutaneous injections of different doses (0.1, 0.14 and 0.2 mg), the average blood concentration of beinaglutide showed a similar trend among different dose groups on different study days. In addition, there was no evidence of beinaglutide accumulation following multiple injections, suggesting the administration plan of injecting beinaglutide three times daily is feasible. These results are consistent with previous studies on beinaglutide (Shanghai benemae pharmaceutical corporation, 2023), providing favourable pharmacological evidence for clinical use. Moreover, the characteristic that distinguishes beinaglutide from other GLP-1RAs is that it has the same amino acid sequence as endogenous GLP-1 in the human body, which is closer to the secretion rhythm of physiological GLP-1 and can effectively simulate the action mode of endogenous GLP-1. Taken together, both the presented PK profile and inherent drug characteristics of beinaglutide supported the suitability of beinaglutide for patients with overweight/obesity to be injected three times daily (5 min before each meals) to weight reduction.

Gender differences analysis indicated that the female participants had higher exposure levels than males, suggesting that the weight reduction effect of beinaglutide in females with overweight/obesity may be better than that in males. This inference was supported by the findings of a phase 3 clinical study using beinaglutide for weight management in Chinese individuals with overweight/obesity (Chen et al., 2024), which showed that female participants in the beinaglutide group had a greater mean weight reduction at week 16 compared to male participants (−6.9% and −4.7%, respectively).

Based on existing studies on the safety of beinaglutide injection in healthy individuals and its effectiveness in T2DM patients, the initial dose of beinaglutide injection for blood glucose control in adult T2DM patients is 0.1 mg. From the perspective of safety and tolerance, this trial selected a lower dose of 0.06 mg as the starting dose for dose escalation, allowing patients to go through a certain period of medication adaptation, reducing the incidence of adverse reactions, improving patient medication safety and adaptability, and then increasing to the clinical starting dose of 0.1 mg. Therefore, the dose of 0.06 mg is only for the medication adaptation period and the PK parameters at this dose level have not been calculated. Considering the small sample size and short period in this trial, it is necessary to study long-term administration in a larger target population to make a recommendation about the medication more accurately.

## 5 Conclusion

Our study provided evidence supporting a favourable safety profile of beinaglutide for the treatment of obesity and overweight, as well as its suitability for weight reduction in overweight/obese populations when administered three times daily. The PK properties of beinaglutide warrant further development and exploration of its clinical applications as an anti-obesity drug.

## Data Availability

The original contributions presented in the study are included in the article/supplementary material, further inquiries can be directed to the corresponding authors.
